# Association of Diaphragmatic Crus Thickness Assessed by Computed Tomography with Diaphragmatic Excursion and Exercise Capacity in Patients with Chronic Obstructive Pulmonary Disease

**DOI:** 10.1007/s00408-026-00915-w

**Published:** 2026-07-21

**Authors:** Masashi Shiraishi, Yuji Higashimoto, Hiroki Mizusawa, Yu Takeda, Masaya Noguchi, Kengo Kanki, Honoka Natsume, Osamu Nishiyama, Ryo Yamazaki, Tamotsu Kimura, Hisako Matsumoto

**Affiliations:** 1 Department of Rehabilitation Medicine, Kindai University Hospital, Faculty of Medicine, Kindai University, 1-14-1 Miharadai, Minami-ku, Sakai, Osaka 590-0197 Japan; 2 Department of Respiratory Medicine and Allergology, Kindai University Hospital, Kindai University Faculty of Medicine, 1-14-1 Miharadai, Minami-ku, Sakai, Osaka 590- 0197 Japan

**Keywords:** Chronic obstructive pulmonary disease, Diaphragmatic crus thickness, Diaphragm, Computed tomography, Ultrasonography, Exercise capacity

## Abstract

**Purpose:**

Diaphragmatic dysfunction contributes to exercise intolerance in chronic obstructive pulmonary disease (COPD). Although diaphragmatic excursion during deep breathing (DE_max_) is associated with exercise capacity, the relationship between computed tomography (CT)-derived diaphragm morphology and diaphragmatic function remains unclear. We investigated the association of CT-derived diaphragmatic crus thickness with diaphragmatic excursion and exercise capacity in COPD.

**Methods:**

This retrospective single-centre study included 100 patients with stable COPD who underwent chest CT, spirometry, ultrasonographic assessment of DE_max_, and 6-minute walk distance (6MWD) testing. Diaphragmatic crus thickness was measured on axial CT images, and the diaphragmatic thickness index (DTI) was calculated after adjustment for body mass index. Multivariable linear regression analyses were performed to identify factors independently associated with DE_max_ and 6MWD. Patients were stratified according to DTI and DE_max_ to evaluate combined structural and functional diaphragm phenotypes.

**Results:**

In multivariable analyses, DTI was independently associated with DE_max_ (β = 0.57, *p* < 0.001) and 6MWD (β = 0.25, *p* < 0.01) after adjustment for age, pulmonary function, and skeletal muscle indices. The combined low-DTI/low- DE_max_ phenotype was independently associated with reduced exercise capacity (6MWD < 350 m; odds ratio 14.89, 95% confidence interval 3.51–87.89, *p* < 0.001).

**Conclusion:**

CT-derived diaphragmatic thickness was independently associated with diaphragmatic excursion and exercise capacity in COPD. Combined structural and functional diaphragm assessment identified patients with markedly reduced exercise capacity, suggesting a role for integrated diaphragm evaluation in functional risk stratification.

**Supplementary Information:**

The online version contains supplementary material available at 10.1007/s00408-026-00915-w.

## Introduction

Chronic obstructive pulmonary disease (COPD) is characterised by persistent airflow limitation and various extrapulmonary manifestations that contribute to exercise intolerance and poor prognosis. Exercise limitation in COPD is multifactorial and involves dynamic hyperinflation, skeletal muscle dysfunction, circulatory impairment, and respiratory muscle dysfunction [1–5]. Among these factors, diaphragmatic dysfunction caused by lung hyperinflation is recognised as a major pathophysiological feature of COPD [6–9]. Hyperinflation leads to diaphragmatic flattening, shortening of muscle fibres, and reduced contractile efficiency, resulting in impaired diaphragmatic function during exercise and daily activities.

Recently, ultrasonography has emerged as a non-invasive and practical method for directly evaluating diaphragmatic function. Diaphragmatic excursion measured by ultrasound has been increasingly used as a functional marker of diaphragmatic performance in chronic respiratory diseases. We previously demonstrated that diaphragmatic excursion during deep breathing (DE_max_) is significantly associated with exercise capacity and dynamic hyperinflation in patients with COPD [10]. Furthermore, we reported that DE_max_ correlates with improvements in exercise tolerance following pulmonary rehabilitation, suggesting that DE_max_ reflects diaphragmatic functional reserve and responsiveness to rehabilitation interventions [11].

In contrast, structural assessment of the diaphragm in COPD remains insufficiently investigated. Computed tomography (CT), which is routinely performed in patients with COPD, enables objective evaluation of diaphragmatic morphology, particularly diaphragmatic crus muscle thickness. A recent CT-based study demonstrated alterations in diaphragmatic morphology, including diaphragmatic crus thickness, in patients with COPD [12]. In single-lung transplant recipients, restoration of normal lung mechanics after transplantation was associated with increased diaphragmatic crus thickness and improved diaphragmatic position, indicating that pulmonary pathology may influence diaphragmatic morphology [13]. However, to our knowledge, no previous study has directly investigated the relationship between ultrasound-assessed diaphragmatic excursion and CT-assessed diaphragmatic crus thickness in patients with COPD.

Clarifying the association between diaphragmatic morphology and diaphragmatic function may improve our understanding of the structural and functional mechanisms underlying diaphragmatic dysfunction in COPD. Furthermore, because chest CT is routinely obtained in clinical practice, CT-based assessment of diaphragmatic morphology may provide a practical and objective surrogate marker of diaphragmatic dysfunction and exercise intolerance. Therefore, the present study aimed to investigate the relationship between diaphragmatic excursion measured by ultrasonography and diaphragmatic crus thickness assessed by CT in patients with COPD. We also explored the association between diaphragmatic crus thickness and exercise capacity.

## Materials and Methods

### Study Design and Participants

This single-centre retrospective cohort study included patients with clinically stable COPD who visited the Department of Respiratory Medicine and Allergology at Kindai University Hospital and were referred for pulmonary rehabilitation between January 2018 and May 2026.

Patients were eligible if they underwent the following assessments within 3 months of DE_max_ measurement: spirometry, chest CT, and exercise tolerance assessment using the 6-minute walk distance (6MWD) test.

Patients with musculoskeletal disorders affecting the diaphragm or skeletal muscles, collagen vascular diseases, and concomitant cerebrovascular diseases were excluded. Because acute exacerbation data could not be reliably collected retrospectively, hospitalisation history between baseline assessment and follow-up CT evaluation was also assessed.

Of the 100 participants included in this analysis, 60 had been included in our previous report [10], whereas the remaining 40 patients were newly enrolled during the extended study period.

### Measurements

Diaphragmatic crus thickness was assessed using axial chest CT images according to a previously validated CT-based measurement method [14]. At the level of the celiac artery origin, the thickness of the right and left diaphragmatic crura was measured at the anterior, middle, and posterior aspects of the vertebral body. Three measurements were obtained from each hemidiaphragm, and the mean of the six measurements was used for subsequent analysis. The mean value of these six measurements was defined as the diaphragmatic crural thickness.

**Fig. 1:**
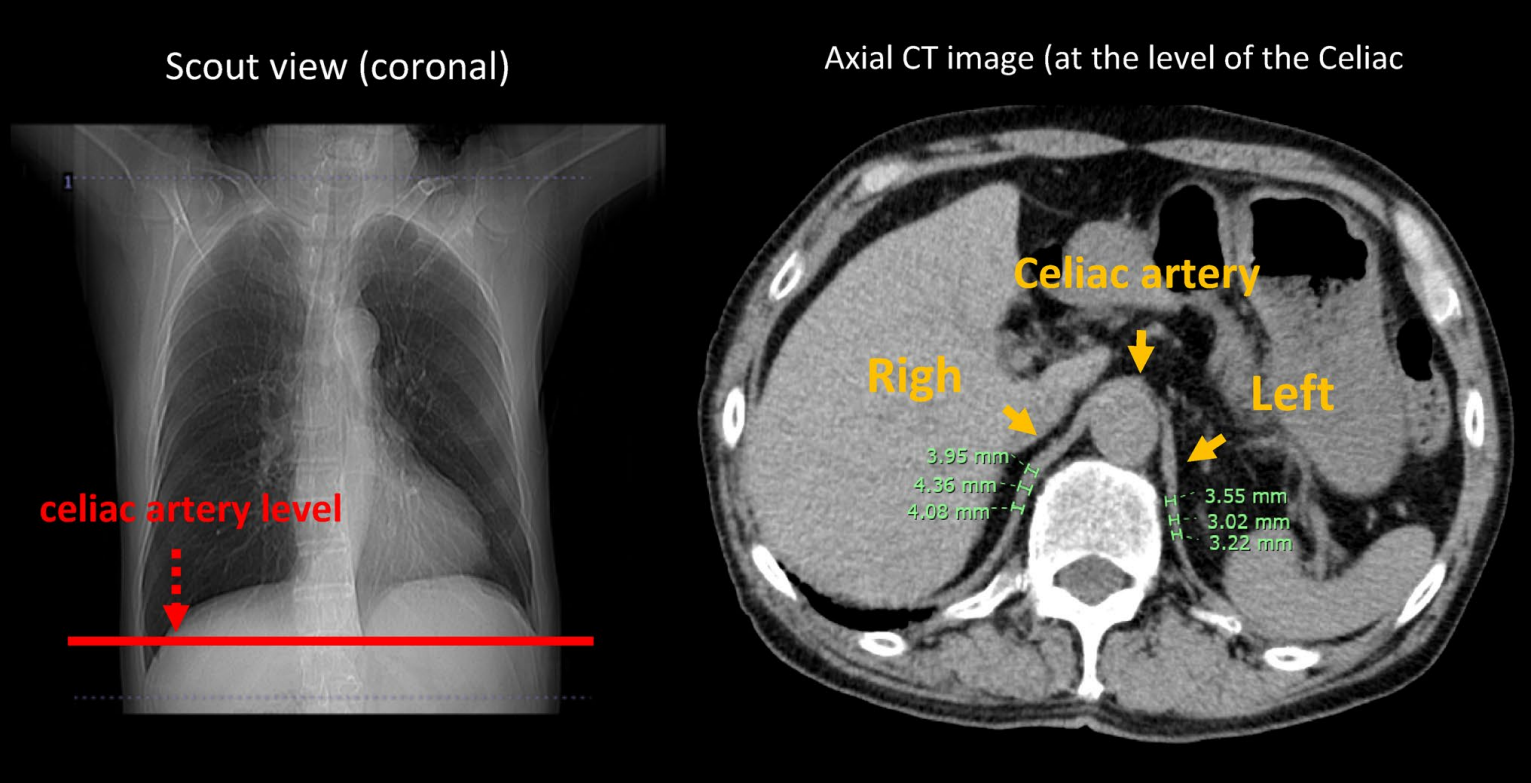
Representative CT image used for the measurement of diaphragmatic crus thickness. Diaphragmatic crus thickness was measured bilaterally at the level of the celiac artery on axial CT images. Measurements were obtained at the anterior, middle, and posterior portions of each crus, and the mean value of six measurements was used for analysis. CT, computed tomography

To adjust for body physique, the diaphragmatic crus thickness index (DTI) was calculated by dividing the mean diaphragmatic crural thickness by body mass index (BMI) and multiplying the value by 100 (Fig. 1) [15].

For quantitative analysis of the erector spinae muscles (ESMs), chest CT images were reconstructed using a mediastinal setting with a reconstruction kernel of FC13. The cross-sectional area of the ESMs was measured on axial CT images using Synapse Vincent software (Fujifilm Medical, Tokyo, Japan). Regions of interest were manually traced at a predefined vertebral level, and the bilateral ESM cross-sectional area was measured according to previously described methods [16, 17]. The sum of the left and right cross-sectional areas was divided by body surface area and expressed as the body surface area adjusted ESM_CSA_.

All CT scans were performed during the stable phase of the disease. Chest CT scans were obtained at full inspiration in the supine position according to the institutional standard protocol [18]. Emphysema was quantified by calculating the percentage of the low attenuation area, defined by the cutoff value of − 950 HU on whole-lung CT images (Aquilion 64 scanner; Toshiba, Tokyo, Japan) using Synapse Vincent (Fujifilm Medical), as previously described [19].

The maximum inspiratory pressure (PI_max_) generated against an occluded airway at the residual volume was measured using respiratory muscle strength testers [20] (IOP-01; Kobata Instrument Manufacturing Ltd., Osaka, Japan).

DE_max_ was measured in the standing position using ultrasonography (Xario 200; Canon Medical Systems, Tokyo, Japan) because exercise capacity and activities of daily living are generally performed in the upright position, allowing diaphragmatic function to be assessed under physiologically relevant conditions. DE_max_ was selected because it reflects diaphragmatic mobility during inspiration and has previously been shown to be associated with exercise capacity in patients with COPD [10, 11]. According to previously described techniques, excursions of the right hemidiaphragm were measured using a 3.5-MHz convex probe [10]. Briefly, the liver was used as an acoustic window, the M-mode cursor was rotated and aligned with the axis of diaphragmatic excursion on the stored image, and the displacement was measured during three deep breaths.

The patients underwent spirometry (CHESTAC-800; Chest, Tokyo, Japan) according to the 2014 American Thoracic Society (ATS) recommendations [21] to measure forced vital capacity (FVC), forced expiratory volume in 1 s (FEV_1_), and inspiratory capacity (IC). The FEV_1_% and FVC% predicted were calculated using the Global Lung Function Initiative method recommended by the 2022 European Respiratory Society (ERS)/ATS technical standard [22] and used by the Japanese Respiratory Society to calculate reference values for spirometry [23].

The 6MWD test was performed to evaluate walking capacity according to the ERS/ATS statement [24–26].

This study was approved by the ethics committee of the Kindai University School of Medicine (approval number R08-016; approved on 27 April 2026). The requirement for informed consent was waived owing to the retrospective nature of the study, and an opt-out approach was used in agreement with the institutional review board.

### Sample Size

The sample size was determined based on the primary analysis of the correlation between DTI and DE_max_. Assuming a moderate correlation coefficient of 0.30, a two-sided α level of 0.05, and a statistical power of 80%, at least 84 patients were required. Considering potential missing data and the planned multivariable analyses, we aimed to include approximately 100 patients with complete datasets.

### Statistical Analyses

To assess the inter-rater reproducibility of CT-derived diaphragmatic crus thickness measurements, two independent evaluators measured the diaphragmatic crus thickness in a randomly selected subset of 40 patients. Inter-rater agreement was evaluated using the intraclass correlation coefficient [ICC (2,1)] and Bland–Altman analysis. Bland–Altman plots were constructed to assess systematic bias and the 95% limits of agreement. Continuous variables are presented as mean±standard deviation. Correlations between DTI and DE_max_, PI_max_, and 6MWD were assessed using correlation analyses. Multivariable linear regression analyses were performed to identify factors independently associated with DE_max_ and 6MWD.

To investigate the combined effects of diaphragmatic structure and function, patients were stratified into four groups according to DTI and DE_max_ values using median cutoffs (HighDTI_HighDE_max_, HighDTI_LowDE_max_, LowDTI_HighDE_max_, and LowDTI_LowDE_max_). Differences in 6MWD among the four groups were analysed using one-way analysis of variance followed by Tukey–Kramer post hoc testing.

Reduced exercise capacity was defined as a 6MWD < 350 m. Logistic regression analyses with likelihood ratio χ² testing were performed to identify factors associated with reduced exercise capacity. Statistical analyses were performed using JMP Pro version 18 (SAS Institute Inc., Cary, NC, USA). A p-value < 0.05 was considered statistically significant.

## Results

**Fig. 2:**
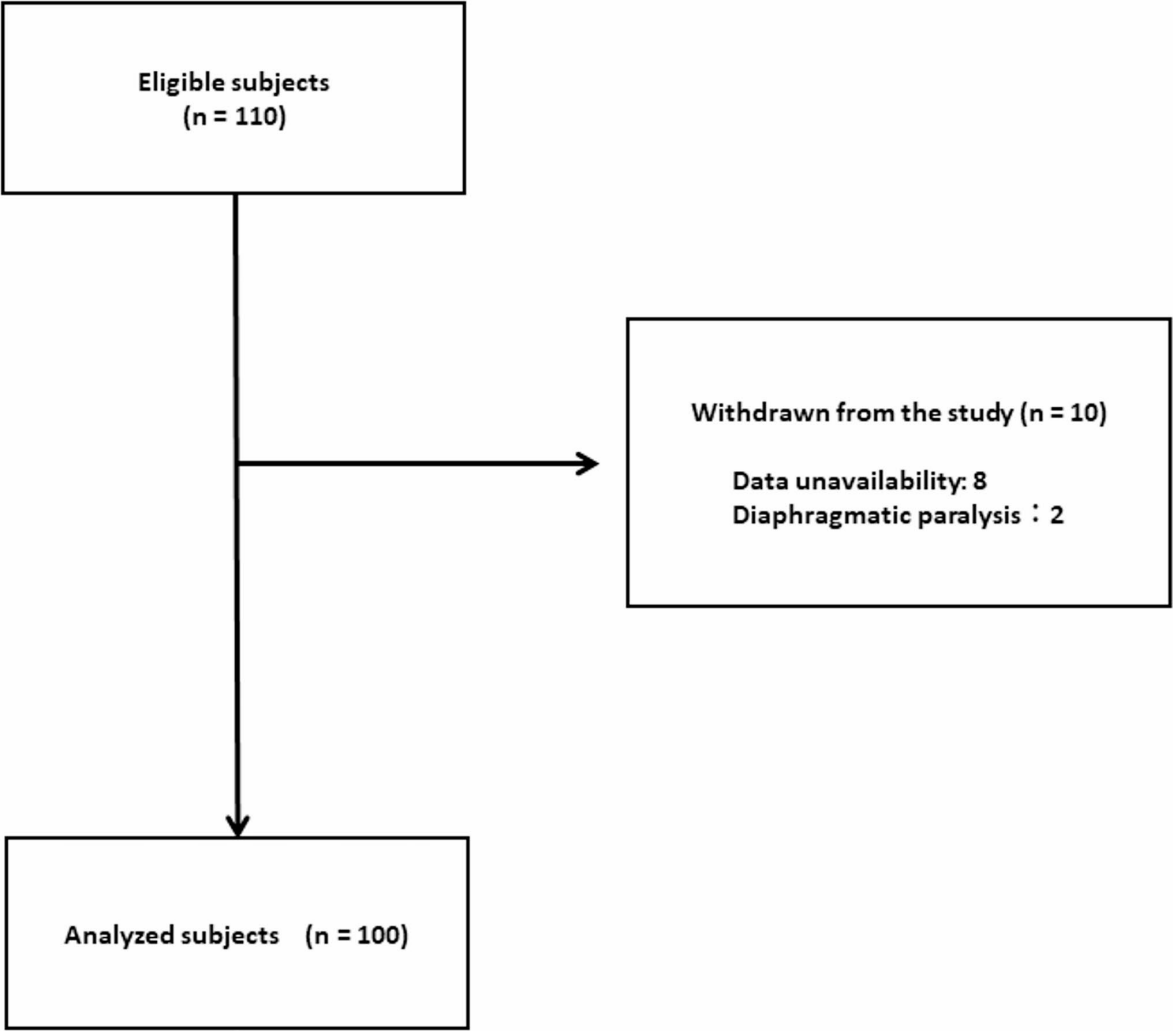
Flow diagram of study participant selection

A total of 110 patients were screened for eligibility. Ten patients were excluded from the final analysis because of unavailable data (*n* = 8) or diaphragmatic paralysis (*n* = 2). Consequently, 100 patients with COPD were included in the final analysis (Fig. 2). No missing data were present among the analysed participants. The baseline characteristics of the study participants are summarised in Table 1. The mean age was 76.4 ± 5.8 years, and the mean FEV₁ % predicted was 56.0%±20.1%. According to the Global Initiative for Chronic Obstructive Lung Disease classification, 7 patients (7%) were classified as stage I, 50 (50%) as stage II, 26 (26%) as stage III, and 17 (17%) as stage IV.

**Table 1:** Baseline characteristics of study participants

Baseline index	*N* = 100
Age, years	76.4 ± 5.8
Body mass index, kg/m²	22.1 ± 3.6
GOLD stage I/II/III/IV, n	7/50/26/17
FEV_1_, L	1.38 ± 0.50
FEV_1_% predicted, %	56.0 ± 20.1
FVC, L	2.94 ± 0.73
FVC, % predicted, %	92.8 ± 19.4
IC, L	2.02 ± 0.54
LAA, %	26.9 ± 14.4
QMS, kgf	29.3 ± 8.1
ESM_CSA_, cm²	22.0 ± 7.3
PI_max_, cmH₂O	57.3 ± 22.1
DE at rest, mm	15.8 ± 3.2
DE_max_, mm	46.9 ± 7.8
DTI	16.3 ± 4.9
CAT score	13.6 ± 6.6
6MWD, m	383 ± 87
Borg dyspnea	4 ± 1
Borg fatigue	3 ± 2

Values are presented as mean ± standard deviation

CAT, COPD Assessment Test; DE, diaphragmatic excursion at rest; DE_max_, maximum diaphragmatic excursion during deep breathing; DTI, diaphragmatic crus thickness index; ESM_CSA_, cross-sectional area of the erector spinae muscle; FEV₁, forced expiratory volume in 1 s; FVC, forced vital capacity; GOLD, Global Initiative for Chronic Obstructive Lung Disease; IC, inspiratory capacity; LAA, low attenuation area; PI_max_, maximal inspiratory pressure; QMS, quadriceps muscle strength; 6MWD, 6-minute walk distance

Inter-rater reproducibility of diaphragmatic crus thickness measurements was good, with an ICC (2,1) of 0.80. Bland–Altman analysis demonstrated minimal systematic bias, and most measurements were distributed within the 95% limits of agreement (Figure S1).

**Fig. 3:**
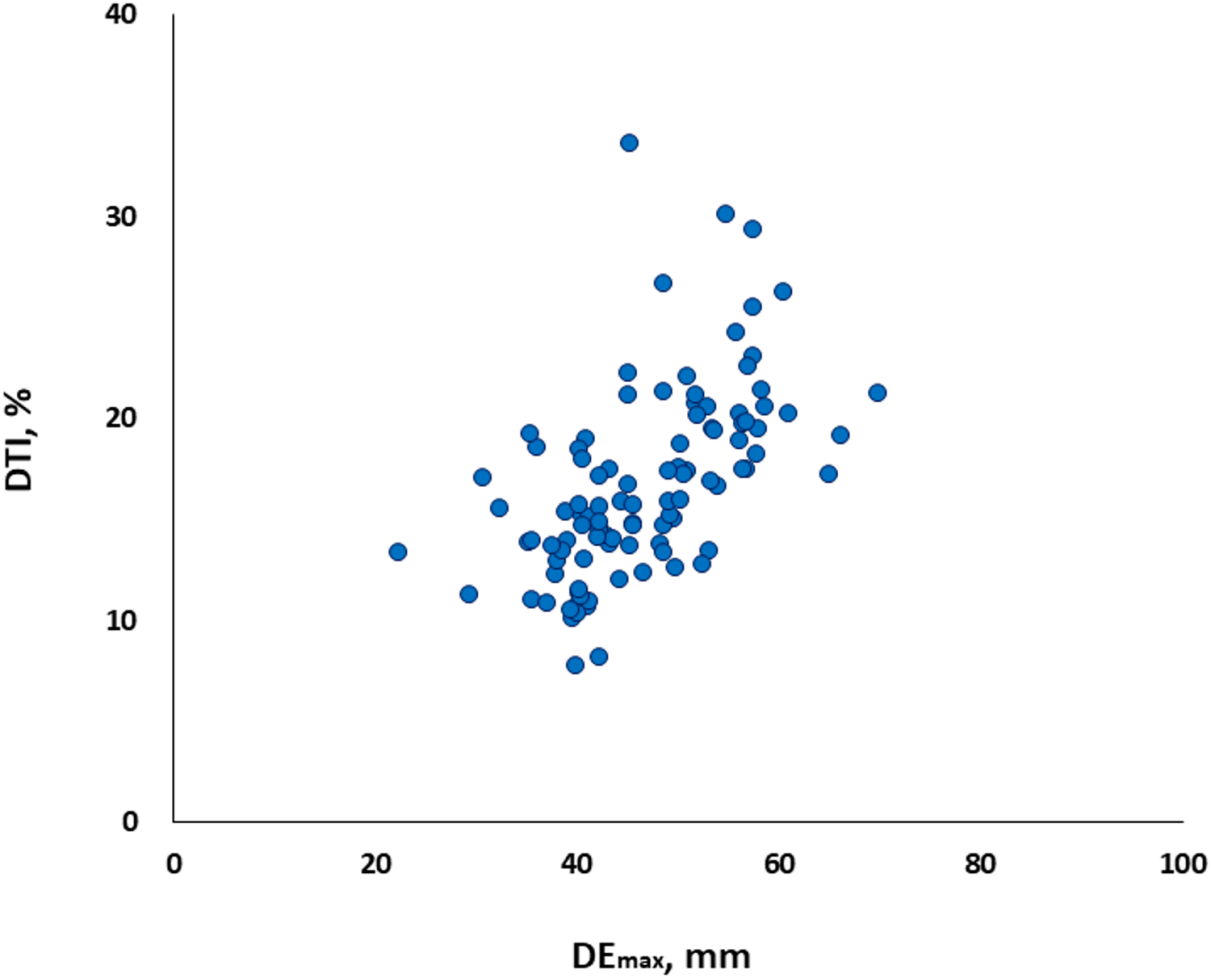
Correlation between DTI and DE_max_. DTI showed a significant positive correlation with DE_max_ (*n* = 100, *r* = 0.61, *p* < 0.001). DE_max_, maximum diaphragmatic excursion during deep breathing; DTI, diaphragmatic crus thickness index

**Fig. 4:**
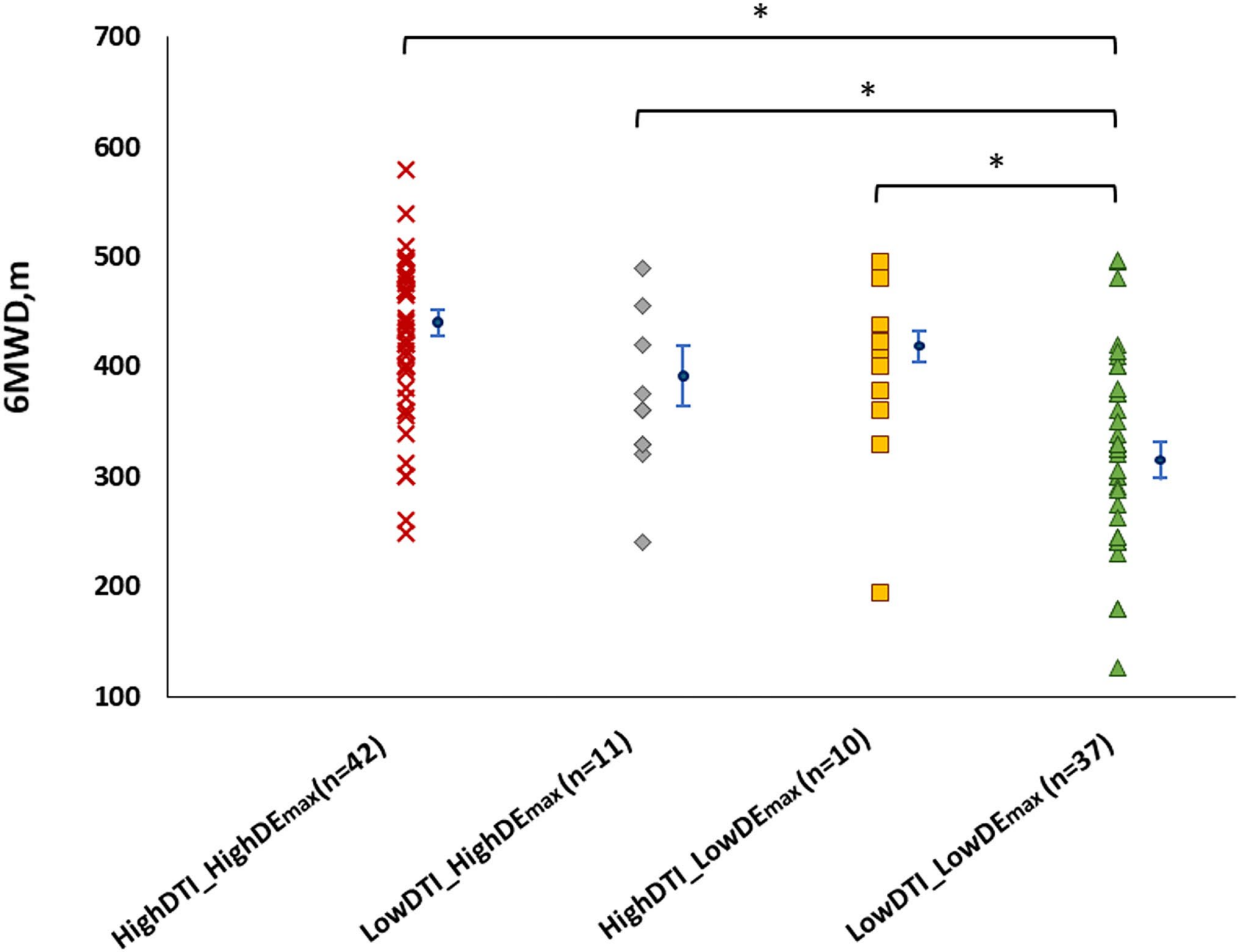
Comparison of 6MWD among the four groups stratified according to DTI and DE_max_. Individual data points are shown, and circles with error bars represent the mean ± standard error. Significant differences in 6MWD were observed among the four groups. The LowDTI_LowDE_max_ group demonstrated the lowest exercise capacity and had significantly lower 6MWD than the HighDTI_HighDE_max_ (*p* < 0.001) and the LowDTI_HighDE_max_ groups (*p* < 0.001). The HighDTI_LowDE_max_ group also showed significantly lower 6MWD than the HighDTI_HighDE_max_ group (*p* < 0.05). DE_max_, maximum diaphragmatic excursion during deep breathing; DTI, diaphragmatic crus thickness index; 6MWD, 6-minute walk distance

DTI showed significant positive correlations with DE_max_ (*r* = 0.61, *p* < 0.001), PI_max_ (*r* = 0.39, *p* < 0.001), and 6MWD (*r* = 0.51, *p* < 0.001) (Fig. 3 and Table S1). In the multivariable linear regression analysis, DTI remained independently associated with DE_max_ after adjustment for age, BMI, pulmonary function, and skeletal muscle indices (β = 0.57, *p* < 0.001) (Table 2). In addition, DTI was independently associated with 6MWD (β = 0.25, *p* < 0.01), together with DE_max_ and FEV_1_ (Table 3). To investigate the combined effects of diaphragmatic structure and function, patients were stratified into four groups according to median cutoff values of DTI (16.3) and DE_max_ (46.9 mm): HighDTI_HighDE_max_ (*n* = 42), LowDTI_HighDE_max_ (*n* = 11), HighDTI_LowDE_max_ (*n* = 10), and LowDTI_LowDE_max_ (*n* = 37) (Fig. 4). Significant differences in 6MWD were observed among the four groups. The LowDTI_LowDE_max_ group demonstrated the lowest exercise capacity and had significantly lower 6MWD than the HighDTI_HighDE_max_ (*p* < 0.001) and LowDTI_HighDE_max_ groups (*p* < 0.001). The HighDTI_LowDE_max_ group also showed significantly lower 6MWD than the HighDTI_HighDE_max_ group (*p* < 0.05).

**Table 2:** Multivariable regression analysis for DE_max_

Independent variable	B	95% CI (B)	β	*p* value	VIF
DTI	1.02	0.78 to 1.25	0.57	*p* < 0.001	1.11
Age, years	− 0.06	− 0.24 to 0.13	− 0.04	0.54	1.19
Body mass index, kg/m²	0.39	0.10 to 0.69	0.18	0.009	1.19
FEV₁, L	0.09	0.04 to 0.15	0.24	*p* < 0.001	1.22
ESM_CSA_, cm²	0.60	0.32 to 0.88	0.30	*p* < 0.001	1.29
QMS, kgf	0.07	− 0.06 to 0.20	0.07	0.30	1.22

B, unstandardized regression coefficient; β, standardized regression coefficient; CI, confidence interval; DE_max_, maximum diaphragmatic excursion during deep breathing; DTI, diaphragmatic crus thickness index; ESMCSA, cross-sectional area of the erector spinae muscle; FEV₁, forced expiratory volume in 1 s; QMS, quadriceps muscle strength; VIF, variance inflation factor; 6MWD, 6-minute walk distance

**Table 3:** Multivariable regression analysis for 6MWD

Independent variable	B	95% CI (B)	β	*p* value	VIF
Age, years	− 2.01	− 4.37 to 0.35	− 0.13	0.10	1.25
Body mass index, kg/m²	3.73	− 0.03 to 7.50	0.15	0.05	1.22
FEV₁, L	0.75	0.02 to 1.48	0.17	*p* < 0.05	1.47
DE_max_, mm	4.22	1.70 to 6.73	0.37	*p* < 0.01	2.69
DTI	5.16	1.41 to 8.90	0.25	*p* < 0.01	1.84
ESM_CSA_, cm²	1.04	− 2.68 to 4.75	0.04	0.582	1.42
QMS, kgf	1.73	0.02 to 3.44	0.15	*p* < 0.05	1.29

B, unstandardized regression coefficient; β, standardized regression coefficient; CI, confidence interval; DE_max_, maximum diaphragmatic excursion during deep breathing; DTI, diaphragmatic crus thickness index; ESMCSA, cross-sectional area of the erector spinae muscle; FEV₁, forced expiratory volume in 1 s; QMS, quadriceps muscle strength; VIF, variance inflation factor; 6MWD, 6-minute walk distance

In the logistic regression analysis using reduced exercise capacity, defined as 6MWD < 350 m, as the dependent variable, DE_max_ was independently associated with reduced exercise capacity, whereas DTI alone was not. However, the combined LowDTI_LowDE_max_ phenotype remained independently associated with reduced exercise capacity after adjustment for age, BMI, FEV₁ and lower extremity muscle strength (odds ratio, 14.89; 95% confidence interval, 3.51–87.89; *p* < 0.001) (Table 4).

**Table 4:** Multivariable logistic regression analysis for reduced exercise capacity (6MWD < 350 m)

Variable	OR	95% CI	*p* value
LowDTI_LowDE_max_ phenotype	14.89	3.51–87.89	*p* < 0.001
Age, years	1.07	0.94–1.24	0.31
Body mass index, kg/m²	0.92	0.74–1.13	0.45
FEV₁, L	0.95	0.93–0.98	*p* < 0.01
QMS, kgf	0.85	0.76–0.93	*p* < 0.01

CI, confidence interval; DTI, diaphragmatic crus thickness index; FEV₁, forced expiratory volume in 1 s; OR, odds ratio; QMS, quadriceps muscle strength; 6MWD, 6-minute walk distance

## Discussion

In the present study, CT-derived DTI was significantly associated with DE_max_, inspiratory muscle strength, and exercise capacity in patients with COPD. Moreover, DTI remained independently associated with both DE_max_ and 6MWD after adjustment for age, BMI, pulmonary function, and skeletal muscle indices. Importantly, the combined LowDTI_LowDE_max_ phenotype was independently associated with reduced exercise capacity, suggesting that structural and dynamic diaphragm assessments provide complementary information regarding functional impairment in COPD.

These findings are biologically plausible because chronic hyperinflation in COPD alters diaphragm geometry and impairs contractile efficiency [27, 28]. Within this context, reduced DE_max_ likely reflects impaired diaphragmatic mobility caused by hyperinflation-related mechanical disadvantage, whereas reduced DTI may reflect structural remodelling or loss of diaphragmatic muscle reserve. The coexistence of both abnormalities may therefore represent more advanced diaphragmatic dysfunction.

Our findings are consistent with our previous ultrasound studies showing that DE_max_ is associated with exercise capacity and dynamic hyperinflation and that improvement in DE_max_ parallels gains in exercise tolerance after pulmonary rehabilitation in patients with COPD [10, 11]. These observations suggest that DE_max_ reflects diaphragmatic functional reserve rather than merely static respiratory mechanics.

The present study extends these observations by demonstrating that CT-assessed diaphragmatic morphology is also associated with diaphragmatic function and ambulatory performance. Because chest CT is routinely performed in COPD management, DTI may provide clinically accessible structural information complementary to ultrasound-derived functional assessment. Furthermore, diaphragm morphology assessed by CT has been linked to airflow limitation and altered respiratory mechanics [18]. Our results further support the concept that structural assessment of the diaphragm may reflect clinically relevant respiratory muscle dysfunction in COPD.

The present findings also fit conceptually with the emerging framework of respiratory sarcopenia, which is characterised by reduced respiratory muscle mass and impaired respiratory muscle strength [29]. Although respiratory sarcopenia is generally evaluated using global respiratory pressure measures such as PI_max_, these measurements do not specifically assess diaphragmatic morphology or motion. In contrast, DE_max_ directly reflects diaphragm-specific movement, whereas DTI may represent structural muscle reserve. DE_max_ reflects diaphragmatic mobility during inspiration and has previously been associated with exercise capacity in patients with COPD.

Therefore, combined assessment using CT-derived DTI and ultrasound-derived DE_max_ may provide a more comprehensive evaluation of diaphragm dysfunction than inspiratory pressure measurements alone.

Although DTI alone was not independently associated with reduced exercise capacity in the multivariable logistic analysis, the combined LowDTI_LowDE_max_ phenotype remained significantly associated with reduced exercise capacity. These findings suggest that structural abnormalities may provide clinically relevant information when interpreted together with dynamic diaphragmatic dysfunction rather than as isolated findings.

From a clinical perspective, integrated diaphragm assessment may help identify patients with poor functional reserve who could benefit from intensified pulmonary rehabilitation, nutritional intervention, or closer evaluation of hyperinflation and respiratory muscle dysfunction. Because both chest CT and diaphragm ultrasonography are increasingly available in routine clinical practice, this combined approach may be feasible in real-world COPD management.

Several limitations should be acknowledged. First, this was a retrospective single-centre study with a relatively modest sample size, which may limit generalisability. Second, DTI is a relatively novel CT-derived parameter, and external validation is required before clinical application. Third, because residual volume and total lung capacity were not available, the relationship among DTI, DE_max_, and static hyperinflation could not be directly evaluated. Fourth, the relatively small subgroup sizes and unequal distribution of phenotype groups may have affected the stability of logistic regression estimates, as reflected by the relatively wide confidence intervals. Finally, this study assessed diaphragm morphology quantitatively using CT but did not include direct measurements of diaphragm contractility or histological assessment of muscle quality.

In conclusion, CT-derived diaphragmatic thickness was significantly associated with diaphragmatic excursion and exercise capacity in patients with COPD. Furthermore, combined reduction in DTI and DE_max_ identified patients with particularly low exercise capacity. Integrating structural and dynamic diaphragm assessments may improve evaluation of respiratory muscle dysfunction and functional risk stratification in COPD.

## Supplementary Information

Below is the link to the electronic supplementary material.

Supplementary Material 1

## Data Availability

The datasets generated and/or analysed during the current study are available from the corresponding author on reasonable request.

## Declarations

### Take-home message

Computed tomography–derived diaphragmatic crus thickness is independently associated with diaphragmatic excursion and exercise capacity in COPD and combined structural and functional diaphragm assessment may improve functional risk stratification.
