# Supplementary material for: Association of Diaphragmatic Crus Thickness Assessed by Computed Tomography with Diaphragmatic Excursion and Exercise Capacity in Patients with Chronic Obstructive Pulmonary Disease
Source: Lung. 2026 Jul 21;204(1):48. doi: 10.1007/s00408-026-00915-w (PMC13388570; doi:10.1007/s00408-026-00915-w)
Supplement: Supplementary file 1 — Supplementary Material 1 [file 408_2026_915_MOESM1_ESM.docx]

**SUPPLEMENTARY INFORMATION**

**Association of diaphragmatic crus thickness assessed by computed tomography with diaphragmatic excursion and exercise capacity**

**in patients with chronic obstructive pulmonary disease**

Masashi Shiraishi^1, *^, Yuji Higashimoto^1^, Hiroki Mizusawa^1^, Yu Takeda^1^, Masaya Noguchi^1^, Kengo Kanki^1^, Honoka Natsume^1^, Osamu Nishiyama^2^, Ryo Yamazaki^2^, Tamotsu Kimura^1^, Hisako Matsumoto^2^

1. Department of Rehabilitation Medicine, Kindai University Hospital, Kindai University Faculty of Medicine, 1-14-1 Miharadai, Minami-ku, Sakai, Osaka 590-0197, Japan
2. Department of Respiratory Medicine and Allergology, Kindai University Hospital, Kindai University Faculty of Medicine, 1-14-1 Miharadai, Minami-ku, Sakai, Osaka 590-0197, Japan

Table S1. Correlations between diaphragm thickness index and clinical variables

| **Variable** | **r** | **95% CI** | **p value** |
| --- | --- | --- | --- |
| Age, years | -0.18 | -0.36 to 0.02 | 0.075 |
| Body mass index, kg/m^2^ | -0.14 | -0.33 to 0.06 | 0.159 |
| FEV_1_ , L | 0.00 | 0.07 to 0.38 | 0.042 |
| FEV_1_ % predicted, % | 0.04 | -0.16 to 0.24 | 0.688 |
| FVC, L | 0.36 | 0.18 to 0.52 | 0.0002 |
| FVC, % predicted, % | 0.16 | -0.03 to 0.35 | 0.11 |
| LAA, % | -0.02 | -0.21 to 0.17 | 0.86 |
| PI_max_, cmH_2_O | 0.39 | 0.21 to 0.54 | <0.0001 |
| DE_max_, mm | 0.61 | 0.48 to 0.72 | <0.0001 |
| QMS, kgf/kg | 0.15 | -0.05 to 0.34 | 0.136 |
| ESM_CSA_, cm^2^ | 0.14 | -0.06 to 0.33 | 0.157 |
| 6MWD, m | 0.51 | 0.35 to 0.64 | <0.0001 |

Values are presented as Pearson’s correlation coefficients (r) with 95% confidence intervals.

DE_max_, maximum diaphragmatic excursion during deep breathing; DTI, diaphragm thickness index; ESM_CSA_, cross-sectional area of the erector spinae muscle; FEV_1_, forced expiratory volume in 1 second; PImax, maximal inspiratory pressure; QMS, quadriceps muscle strength; 6MWD, 6-minute walk distance.

Figure S1. Bland–Altman plot for inter-rater agreement of CT-derived diaphragmatic crus thickness measurements.

A and B indicate measurements independently performed by two raters (MS and KK).

The solid red line indicates the mean difference (bias), and the dashed lines indicate the 95% limits of agreement.
